# Aromatase Inhibitors for Endometriosis-Associated Infertility; Do We Have Sufficient Evidence?

**DOI:** 10.22074/ijfs.2016.5040

**Published:** 2016-09-05

**Authors:** Hatem Abu Hashim

**Affiliations:** Department of Obstetrics and Gynecology, Faculty of Medicine, Mansoura University, Mansoura, Egypt

**Keywords:** Endometriosis, Infertility, Aromatase, Assisted Reproductive Technology

## Abstract

Orally active aromatase inhibitors (AIs) have gained attention for treatment of infertile women with endometriosis in whom aromatase p450 is aberrantly expressed. This review aimed to critically appraise and summarize the available evidence concerning the use of AIs for management of endometriosis-associated infertility. PubMed was searched to May 2015 with the following key words: endometriosis, infertility and aromatase. Priority was given for randomized controlled trials (RCTs) followed by other study designs. Main outcome measures were as follows: rates of clinical pregnancy, miscarriage and live birth as well as endocrine outcomes. Eighty-two abstracts were screened and six original articles were included. A RCT demonstrated that post-operative letrozole treatment did not improve spontaneous pregnancy rate. Another RCT reported no superiority of letrozole superovulation over clomiphene citrate (each combined with intrauterine insemination) in minimalmild endometriosis and previous laparoscopic treatment. Anastrozole significantly inhibited the growth of endometriotic cells and their estrogen production in culture. In assisted reproductive technology (ART) cycles, dual suppression (Agonist/anastrozole) was tested in a pilot study with a pregnancy rate of 45% however, high pregnancy loss (30%) occurred. A retrospective study showed that letrozole may improve endometrial receptivity in endometriotic patients undergoing *in vitro* fertilization (IVF). An opposite view from an *in vitro* study showed lower estradiol production and aromatase expression in cultured granulosa cells from endometriotic women undergoing IVF and marked reduction under letrozole. In conclusion, current evidence is limited. More trials are warranted to enhance our knowledge and provide a clear and unequivocal evidence to guide our clinical management of infertile women with endometriosis using AIs.

## Introduction

**Fig.1:**
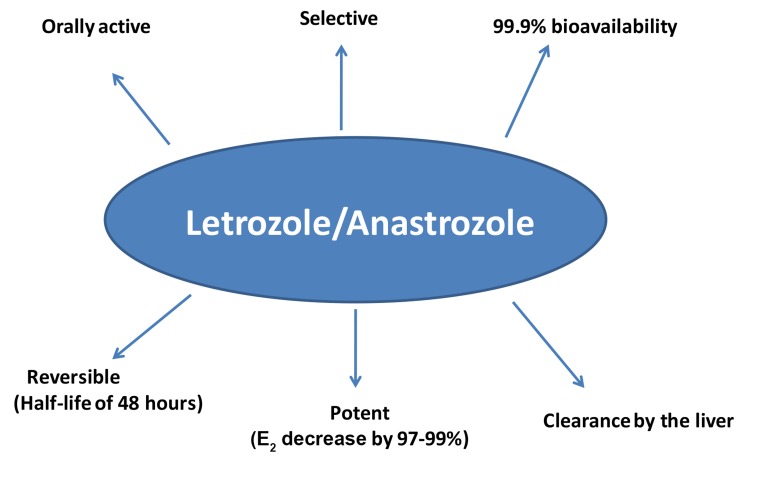
Favorable pharmacokinetics and pharmacodynamics of letrozole and anastrozole.

Endometriosis is a complex clinical issue, in which the quote of Albert Einstein "The most incomprehensible thing of the world is that it is comprehensible” is merit worthy. In this context, endometriosis is not only classically defined as the presence of endometrial glands and stroma outside the uterine cavity (1), but also well known as three separate entities (peritoneal, ovarian, or deeply infiltrating) (2) as well as 4 distinct stages (from minimal or stage 1 to severe disease or stage 4) (3). Nevertheless, endometriosis remains enigmatic in its pathogenesis and controversial in therapy till now (4). It is estimated that 5 to 10% of reproductive age women have endometriosis with a higher prevalence rate up to 50% among infertile women (5). Importantly, endometriosis-associated infertility represents a therapeutic dilemma because of its illusive background including ovulatory dysfunction related to altered folliculogenesis as well as impaired steroidogenesis of granulosa cells, impaired fertilization, low quality embryos, defective implantation, sperm phagocytosis, the embryo toxic environment and pelvic adhesions in advanced stages (6). Aromatase p-450 enzyme is the key enzyme in the biosynthesis of estradiol (E2) in the ovarian granulose cells of premenopausal women. Notably, unlike women without endometriosis, high levels of aromatase P450 enzyme expression has been shown in eutopic endometrial tissue as well as in ectopic endometrial implants in endometriotic patients (7). This abnormal aromatase expression results in local estrogen production by endometriotic implants. Together with other mechanisms such as altered immune responses, angiogenesis and apoptosis, the autonomously produced estrogen leads to inflammation, proliferation and survival of endometriotic implants (8-10). In view of their ability to inhibit estrogen biosynthesis, third generation aromatase inhibitors (AIs) mainly letrozole and anastrozole have challenged clomiphene citrate (CC) in management of infertile patients with polycystic ovary syndrome as well as those with unexplained infertility (11). Additionally, the use of AIs to suppress the locally produced E_2_ by endometriotic deposits has gained ground as a potential therapy for correcting abnormal endocrine and reproductive function of patients with endometriosis (9-11). Noteworthy, letrozole and anastrozole have favorable pharmacokinetics and pharmacodynamics facilitating their use in clinical practice due to the following reasons: being selective, completely absorbed orally with absolute bioavailability of 99.9%, reversible with mean halflife of 48 hours, greater potency with reduction in serum estrogen by 97 to 99% and clearance mainly by the liver (Fig .1) (12). In that regard and given that this is a clinically important area to be addressed, this review was conducted to critically appraise and summarize the current evidence concerning the use of AIs for management of endometriosis-associated infertility in both non-assisted as well as assisted reproductive technology (ART) cycles.

Eighty-two abstracts were retrieved from an electronic search using the PubMed from inception to May 15, 2015 with the following search terms "endometriosis", "infertility" and "aromatase" without search filters. Studies in which reproductive outcome (rates of clinical pregnancy, miscarriage and live birth) as well as endocrine outcomes were included in the following order systematic reviews and meta-analyses of randomized controlled trials (RCTs), RCTs, prospective cohort studies and other observational studies. Manual screening of references of the retrieved articles was done to identify other pertinent studies. Finally, results of 6 original articles were included concerning the use of AIs for management of endometriosis-associated infertility in both non-assisted as well as ART cycles (Tables1, 2) (13-18).

**Table 1:** Aromatase inhibitors in endometriosis-associated infertility in non-ART cycles


References	Study design/sample size	Concept	Intervention	Outcome

Alborzi et al. (13)	RCT (n=144)	Post-operative suppression with letrozole and pregnancy outcome as well as the disease recurrence rate	Letrozole 2.5 mg/day vs. triptorelin 3.75 mg IM every month vs. no medication for 2 months after laparoscopic surgery, with a 12 months follow up	No significant differences among the three groups with regard to the pregnancy rate (23.4 vs. 27.5 and 28.1% respectively) as well as the disease recurrence rate
Abu Hashim et al. (14)	RCT (n=136)	Superovulation with letrozole+IUI in stage I-II endometriosis with no pregnancy 6-12 months after laparoscopy	Letrozole/IUI vs. CC/IUI	No significant differences between both groups for clinical pregnancy rate per cycle, cumulative pregnancy rate, miscarriage, or live birth rates.


ART; Assisted reproductive technology, CC; Clomiphene citrate, IM; Intramuscular injection, IUI; Intrauterine insemination, and RCT; Ran- domized controlled trial.

**Table 2:** Aromatase inhibitors in women with endometriosis-associated infertility undergoing ART


References	Studydesign/sample size	Concept	Intervention	Outcome

Badawy et al. (15)	An *in vitro* study on cultured endometriotic cells	To demonstrate the effect of anastrozole, on the growth and E_2_ production of endometriotic cells in culture	First addition of testosterone (10 µg/mL) to the culture medium then addition of anastrozole, in a dose of 200 µg/mL and 300 µg/mL,	Anastrozole produced significant decrease in endometriotic cell count as well as decrease in E_2_ secretion and this effect was dose dependent.
Lossl et al. (16)	A prospective pilot study [n=20 with endometriomas undergoing IVF (n=16)/ICSI (n=4)]	Dual suppression	Prolonged down-regulation by combined 3-month GnRHa^+1^ mg anastrozole/day prior to IVF	Significant reduction of endometriomal volume (29%) and serum CA125 (61%). 45% clinical pregnancy rate and 15% live birth rate.
Miller et al. (17)	A retrospective cohort study (n=97 with endometriosis undergoing IVF)	Letrozole co-treatment might improve the IVF success rates by improving endometrial receptivity	29/79 women undergoing stand- ard IVF lacked normal integrin expression. Other 18 integrinnegative women received letrozole early in IVF stimulation (5 mg, days 2-6).	Significantly higher clinical pregnancy and delivery rates observed in integrin-negative patients who received letrozole as compared to those who did not receive letrozole (61 vs. 14%, P<0.001 and 50 vs. 7%, P<0.001, respectively)
Lu et al. (18)	An in vitro study on cultured LGC	Letrozole may compromise aromatase activity of LGC resulting in a poor reproductive outcome in patients with stage III/IV endometriosis undergoing ART	Effect of different concentrations of letrozole on E_2_ production and P450 aromatase mRNA expression in cultured LGC from women with (n=23) and without endometriosis (n=19)	Significantly lower E_2_ production and P450 aromatase mRNA expression occurred in women with endometriosis and further reduction of these parameters were demonstrated following letrozole in a con- centration of 1 µmol/L.


ART; Assisted reproductive technology, CA; Cancer antigen, E2 ; Estradiol, GnRHa; Gonadotropin-releasing hormone agonist, ICSI; Intracytoplasmic sperm injection, IVF; *In vitro* fertilization, and LGC; Luteinized granulosa cells.

## Discussion

### The postoperative use of aromatase inhibitors in women who underwent laparoscopic surgery for endometriosis-associated infertility

The recent European Society of Human Reproduction and Embryology (ESHRE) Endometriosis Guideline demonstrated no evidence to support the use of postoperative hormonal therapy to improve spontaneous pregnancy rates in infertile women with endometriosis (19). This recommendation was based on the findings of a Cochrane metaanalysis by Furness et al. (20) including eight studies with 420 patients with endometriosis-associated infertility who were treated postoperatively by different modalities such as gonadotropin-releasing hormone agonist (GnRHa), medroxyprogesterone acetate, danazole and gestrinone [risk ratio (RR)=0.84, 95% confidence intervals (CI): 0.591.18]. Does the postoperative use of AIs increase the spontaneous pregnancy rate in women with endometriosis-associated infertility? This is a very relevant clinical question. Noteworthy, only one prospective RCT by Alborzi et al. (13) addressed this point among 144 patients who were diagnosed to have different stages of endometriosis ranging from minimal to severe disease by laparoscopy and histological confirmation. Patients were randomly allocated into the three following groups: group 1 who received letrozole 2.5 mg/day (n=47 cases), group 2 who had triptorelin (GnRHa) 3.75 mg intramuscular (IM) injection every 4 weeks (n=40 patients) and group 3 who received no medication for 2 months after laparoscopic surgery (n=57 patients) with a 12 months follow up period. The authors reported no significant differences among the three groups with regard to the pregnancy rate (23.4% in group 1 vs. 27.5% and 28.1% in groups 2 and 3 respectively) as well as the disease recurrence rate defined by recurrent symptoms in the form of dysmenorrhea, dyspareunia and pelvic pain (6.4% in group 1 vs. 5% and 5.3% in groups 2 and 3 respectively). Therefore, the authors did not recommend the post-operative use of letrozole or GnRHa in women undergoing surgery for endometriosis-associated infertility (13). The finding from this RCT is in agreement with the aforementioned lack of beneficial effect of postoperative hormonal therapy on endometriosis-associated infertility (19). 

### Superovulation with aromatase inhibitors combined with intrauterine insemination for women with minimal or mild endometriosis-associated infertility 

In view of the recent ESHRE Endometriosis Guideline, superovulation with intrauterine insemination (IUI) may be effective in increasing live birth rate, compared with expectant management in women with minimal to mild endometriosis (19,21). In addition, superovulation with IUI may be more effective in increasing pregnancy rate than IUI alone and may be as effective in women with minimal or mild endometriosis within 6 months of surgical treatment as in women with unexplained infertility. In these women, there are potential benefits of superovulation including, improvement of the endocrine environment, correction of subtle ovulatory dysfunction as well as an increase in the number of mature oocytes. Thereby, it optimizes the chance per cycle of fertilization, embryo development and implantation (19,22,23). Unlike CC, letrozle has no downstream effects on the endometrium and cervical mucus. This could be attributed not only to its relatively shorter half-life (~2 days vs. ~2 weeks in CC), but also for its peculiar mechanism of action via down regulation of the E_2_ synthesis rather than competitive inhibition of its action (11,12). Furthermore, in ovulatory infertile patients, letrozole was reported to induce moderate ovarian hyperstimulation with E_2_ levels similar to spontaneous cycles and higher midluteal progesterone, resulting in a normal endometrial histology and development of pinopodes, which could increase endometrial receptivity (24). Thereby, the biologic plausibility of the concept that superovulation with letrozole has the potential to increase pregnancy in women with minimal or mild endometriosis undergoing IUI is attractive, but is it clinically relevant? 

In line with this concept, Abu Hashim et al. (14) in a RCT compared pregnancy rates following superovulation between letrozole and CC (each combined with IUI). The study included 136 women with primary infertility and no pregnancy following laparoscopic treatment for minimal to mild endometriosis over a 6-12-month follow-up period. Women were allocated to receive either 5 mg letrozole/day (69 women, 220 cycles) or 100 mg CC/day (67 women, 213 cycles) for 5 days, combined with IUI for up to 4 cycles. The authors demonstrated that the total number of follicles and serum E_2_ on the day of hCG administration were significantly higher in the CC group; however, no significant differences were found with regard to the clinical pregnancy rate per cycle or the cumulative pregnancy rate after 4 cycles in both groups (15.9 vs. 14.5% and 64.7 vs. 57.2% in letrozole and CC groups, respectively). No significant differences were also found in miscarriage and live birth rates between both groups (11.4 vs. 12.9% and 44.9 vs. 40.3% in letrozole and CC groups, respectively). The authors concluded that superovulation with letrozole is not more effective than CC combined with IUI for women with minimal to mild endometriosis who did not achieve pregnancy after 6-12 months following laparoscopic treatment (14). 

### The effect of endometriosis on in vitro fertilization outcome

In a recent meta-analysis, Harb et al. (25) examined the effect of endometriosis on *in vitro* fertilization (IVF) outcome in twenty-seven observational studies that included 8984 women. The authors demonstrated that the presence of severe endometriosis (stage III/IV), but not minimalmild endometriosis (stage I/II), was associated with reduced the implantation rate (RR=0.79, 95% CI: 0.67-0.93, P=0.006) and clinical pregnancy rate (RR=0.79, 95% CI: 0.69-0.91, P=0.0008) as compared to those of women with other causes of infertility. This finding may be attributed to defective embryo quality as well as defective endometrial receptivity (26,27). High endometrial aromatase P450 mRNA expression in endometriotic women was also reported to be associated with poor IVF outcome (28). On the other hand, a reduction in fertilization rates was demonstrated in stage I/II endometriosis but not in stage III/IV disease as compared to controls (RR=0.93, 95% CI: 0.87-0.99, P=0.03). This interesting finding may be partially explained by impaired steroidogenesis of granulosa cells as well as poor oocyte quality in stage I/II disease (6). It is noteworthy that both of the third-generation AIs, letrozole and anastrazole, have been used as adjuvant treatments in ART that is mainly for poor responders based on their ability to increase follicular sensitivity to follicle stimulating hormone (FSH) as a result of increased intrafollicular androgens (29). This point is outside the scope of this review. 

### The single and dual suppression concepts in women with endometriosis undergoing assisted reproductive technology 

The merits of pituitary down-regulation for 3-6 months with a GnRHa in women with endometriosis before ART were previously demonstrated (30). Furthermore, this practice was recommended in the updated ESHRE Endometriosis Guideline (19). This recommendation was based on the findings of a Cochrane meta-analysis of three RCTs including 165 patients who had endometriosisassociated infertility (31). The authors concluded that this suppressive policy prior to ART increases the odds of clinical pregnancy by more than 4-fold (OR=4.28, 95% CI: 2.00-9.15, P=0.0001). This beneficial effect may be explained by increased activity of the immune system [increased natural killer (NK) cells, decreased auto-antibodies levels and reduction of elevated levels of peritoneal fluid interleukin-1 (IL-1) and tumour necrosis factor alpha (TNF-α), stimulation of endometrial cell apoptosis as well as improved endometrial receptivity and implantation rates (30,32). The latter action may be due to the suppression of endometrial aromatase expression by GnRHa (33). The concept of dual suppression (GnRHa+AI) prior to IVF has an appealing biological plausibility through the additional blockade of extraovarian aromatase enzyme aberrantly expressed in endometriotic implants as well as the eutopic endometrium of women with endometriosis (9,11). A more recent *in vitro* study demonstrated that anastrozole significantly suppressed endometriotic cells proliferation in culture as a result of marked reduction in the E_2_ levels in these cells and this effect was dose dependent i.e. the suppression of growth and proliferation of the cultured endometriotic cells increased following increased anastrozole dosage (15). Thereby, dual suppression prior to IVF may result in a higher pregnancy rate rather than suppression with GnRHa alone. In a prospective pilot study, Lossl et al. (16) tested this concept among 20 infertile patients with endometriomas undergoing IVF/ Intracytoplasmic sperm injection (ICSI). A 3-month GnRHa course (3.6 mg goserelin administered through a subcutaneous injection on the treatment days 1, 28 and 56) combined with anastrozole (1 mg daily from the days 1 to 69) was used followed by controlled ovarian stimulation from the day 70. Interestingly, the authors demonstrated that prolonged dual suppression significantly reduces endometriomal volume by 29% (3-39%, P=0.007) and serum CA125 by 61% (21-74%, P=0.001). Nine of the 20 patients (45%) achieved pregnancy; however, only 3 patients (15%) delivered (two singletons and one twin). The authors speculated that the high pregnancy loss (6 patients) could be accidental or due to impaired quality of the oocytes and/or the endometrium (16). So far, there are no RCTs available in the literature comparing the use of goserelin+anastrozole suppression with single suppression by the GnRHa alone. 

### Aromatase inhibitors and endometrial receptivity in endometriotic women undergoing assisted reproductive technology 

As endometrial aromatase expression was found to be associated with poor IVF outcome (28), it is theoretically plausible that the use of AIs in endometriotic women may be advantageous in improving their endometrial receptivity and the IVF outcome. Of note, reduced integrin expression, a marker of endometrial receptivity, has been shown in women with IVF failure (34). In a retrospective cohort study, Miller et al. (17) evaluated the effect of letrozole on endometriotic infertile women undergoing IVF and lacking normal integrin expression assessed by immunohistochemistry in mid-luteal endometrial biopsies. Out of 79 women undergoing standard IVF, 29 (36.7%) lacked normal integrin expression. Meanwhile other 18 integrin-negative women received letrozole early in IVF stimulation (5 mg, days 2-6). Notably, in women undergoing standard IVF, clinical pregnancy and delivery rates were significantly higher in women with normal integrin expression compared with integrin-negative women [20/50 (40%) vs. 4/29 (13.7%), P=0.02 and 19/50 (38%) vs. 2/29 (7%), P<0.01, respectively]. Additionally, among integrin-negative patients, significantly higher clinical pregnancy and delivery rates occurred in those who received letrozole as compared to women who did not receive letrozole [11/18 (61%) vs. 4/29 (14%), P<0.001 and 9/18 (50%) vs. 2/29 (7%), P<0.001, respectively]. The authors concluded that letrozole co-treatment might improve the IVF success rates in a subset of women with endometriosis and implantation failure. However, they admitted the need for further prospective studies to confirm these findings (17). 

### The use of aromatase inhibitors in endometriotic women undergoing assisted reproductive technology; a counterintuitive view 

Compared to women with tubal or unexplained infertility undergoing ARTs, reduced aromatase activity in granulosa cells as well as reduced expression of the cytochrome p450 family 19 subfamily A member 1 (*CYP19A1*) gene (that encodes aromatase) in cumulus cells has been shown in infertile women with endometriosis by *in vitro* studies (35,36). Thereby, defective granulosa cell steroidogenesis could explain the impaired oocyte quality and the reduced fertilization rate associated with endometriosis. In that regard, an opposite view is that the use of AIs in patients with endometriosis undergoing ART may further compromise aromatase activity of granulose cells ending with a poor reproductive outcome. This possibility has been tested recently by Lu et al. (18) who compared E_2_ production and P450 aromatase mRNA expression of cultured luteinized granulosa cells and the effect of letrozole on these parameters between women with (n=23) and without endometriosis (n=19). Of note, significantly lower E_2_ production and P450 aromatase mRNA expression occurred in women with endometriosis and further reduction of these parameters were demonstrated following letrozole in a concentration of 1 µmol/L. Importantly, the results of this study should be interpreted with caution because it included women with advanced stage endometriosis (III-IV). So, whether early stage endometriosis (I-II) has a similar effect on *in vitro* E_2_ production and P450 aromatase mRNA expression remains uncertain. The authors admitted the need for larger *in vivo* studies for further validation of these findings (18). 

### Safety of aromatase inhibitors

Data from the only abstract that did report increased rates of cardiac and bone malformations with letrozole treatment for infertility were subsequently unsubstantiated in lager trials (37,38). More recently, in a large double-blind, multicenter RCT, Legro et al. (39) found no significant difference in the anomaly rates in infertile women with polycystic ovary syndrome who conceived with CC or letrozole (1.5 vs. 3.9%, respectively) and even these rates appear similar to that reported in healthy fertile women who conceived without undergoing ART(5.8%) (40). 

## Conclusion

In women with endometriosis, aromatase p450, the key enzyme in the biosynthesis of E_2_ is not only present in the ovarian granulosa cells but also aberrantly expressed in the eutopic endometrium as well as in endometriotic deposits. Thereby, the orally active third-generation AIs letrozole and anastrozole have gained attention as a cotreatment for endometriosis associated infertility. In non-ART cycles, a RCT has demonstrated that post-operative treatment with letrozole or GnRHa for 2 months did not improve spontaneous pregnancy rates as compared to no treatment. Another RCT reported no superiority of superovulation with letrozole over CC followed by IUI in women with minimal-mild endometriosis who have not achieved pregnancy 6-12 months following laparoscopic treatment. Anastrozole significantly inhibited the growth of endometriotic cells and estrogen production in culture was also dose-dependent. In ART cycles, the concept of dual suppression (3-month GnRHa+1 mg anastrozole/day) was tested in a pilot study in 20 infertile women with endometriomas undergoing IVF. A significant reduction in endometrioma volume and serum CA-125 concentration was found. Of note, although the pregnancy rate was 45%, a high pregnancy loss was observed and the live-birth rate was 15%. So far, this concept has not been compared with single suppression by the agonist alone. Evidence from a retrospective cohort study has shown that letrozle could potentially improve endometrial receptivity in endometriosis patients undergoing IVF in whom aromatase is aberrantly expressed. On the other hand, an *in vitro* study reported lower E_2_ production and aromatase expression of cultured granulosa cells in women with stage III-IV endometriosis undergoing IVF. Letrozole further reduced these parameters. Importantly, these findings should be interpreted with caution. No further validation has been made available so far in *in vivo* studies. Overall, in view of the current limited body of evidence of 6 original studies, the research agenda for the future should address more questions concerning the use of AIs in patients with endometriosis such as the ideal timing for AIs cotreatment, optimum dosage, and definite effect on endometrial receptivity. In that respect, the quote of Albert Einstein "Learn from yesterday, live for today, hope for tomorrow. The important thing is not to stop questioning” is merit worthy as evidence-based medicine is an evolving process. So, more trials are still needed to have a clear and unequivocal evidence to guide our clinical practice and to help achieve best outcomes. 
